# How to identify, incorporate and report patient preferences in clinical guidelines: A scoping review

**DOI:** 10.1111/hex.13099

**Published:** 2020-07-12

**Authors:** Claire Kim, Melissa J. Armstrong, Whitney B. Berta, Anna R. Gagliardi

**Affiliations:** ^1^ University Health Network Toronto ON Canada; ^2^ University of Florida Gainesville FL USA; ^3^ University of Toronto Toronto ON Canada

**Keywords:** patient participation, patient‐centred care, practice guidelines as topic, quality improvement

## Abstract

**Background:**

Clinical guidelines optimize care delivery and outcomes. Guidelines support patient engagement and adherence if they reflect patient preferences for treatment options, risks and benefits. Many guidelines do not address patient preferences. Developers require insight on how to develop such guidelines.

**Objective:**

To conduct a scoping review on how to identify, incorporate and report patient preferences in guidelines.

**Search:**

We searched MEDLINE, EMBASE, Scopus, CINAHL, OpenGrey and GreyLit from 2010 to November 2019.

**Eligibility:**

We included English language studies describing patient preferences and guidelines.

**Data extraction and synthesis:**

We reported approaches for and determinants and impacts of identifying patient preferences using summary statistics and text, and interpreted findings using a conceptual framework of patient engagement in guideline development.

**Results:**

Sixteen studies were included: 2 consulted patients and providers about patient engagement approaches, and 14 identified patient preferences (42.9%) or methods for doing so (71.4%). Studies employed single (57.1%) or multiple (42.9%) methods for identifying preferences. Eight (57.1%) incorporated preferences in one aspect of guideline development, while 6 (42.9%) incorporated preferences in multiple ways, most commonly to identify questions, benefits or harms, and generate recommendations. Studies did not address patient engagement in many guideline development steps. Included studies were too few to establish the best approaches for identifying or incorporating preferences. Fewer than half of the studies (7, 43.8%) explored barriers. None examined reporting preferences in guidelines.

**Conclusions:**

Research is needed to establish the single or multiple approaches that result in incorporating and reporting preferences in all guideline development steps.

## 1 BACKGROUND

Clinical guidelines are a fundamental approach for translating knowledge to policy and practice because they synthesize the totality of evidence on a given condition, disease, procedure or therapy, and provide recommendations that support decision‐making for health‐care planning, delivery, evaluation and improvement.^1^ Guidelines have long been recognized as a means of improving health‐care professional behaviour and clinical outcomes across all conditions and settings of care.^2^, ^3^ Apart from directly informing practice, guideline recommendations can be embedded in clinical decision support applications^4^ or inform the development of clinical pathways that facilitate multidisciplinary teamwork^5^ or performance measures that underpin evaluation and quality improvement efforts.^6^

Considerable research over many decades has generated insight on how to actively implement guidelines by pre‐identifying potential multi‐level barriers of use^7^ and using that information to choose and tailor implementation strategies from among a plethora of educational, social, organizational and system‐level options.^8^, ^9^ In concert with implementation strategies, another important strategy for supporting guideline adoption is to ensure that guidelines are implementable, referring to the characteristics of guideline that help end‐users apply them.^10^ For example, guideline developers can employ the Appraisal of Guidelines for Research and Evaluation (AGREE) Consortium, Institute of Medicine, and Grading of Recommendations Assessment, Development and Evaluation processes to ensure that guidelines clearly describe methods by which they were developed, evidence upon which they were based and recommendations for practice, which all contribute to guideline implementability.^11^, ^12^, ^13^ Guidance is also available for developing implementation tools that can be included in or with guidelines^14^, ^15^, ^16^ and are proven to facilitate guideline uptake by clinicians.^17^

Another way to render guidelines implementable is to formulate the recommendations based on patient preferences and offer guidance to clinicians on how to address patient preferences when applying the recommendations. Patient preferences, defined broadly as ‘the desire for specific satisfiers of basic needs’ and referring to perspectives, values or priorities related to health and health care, are associated with patient satisfaction with health‐care experiences.^18^ Patient preferences, collected through means such as interviews, focus groups or questionnaires, or by including patients on guideline development panels, can influence guidelines in many ways. For example, patients articulated concerns unique from clinicians on renal disease lifestyle, psychosocial support, quality of life and outcomes, which shaped the development of a guideline on polycystic kidney disease,^19^ and patient value judgments about outcomes associated with palliative chemotherapy influenced panel discussions and informed recommendations in six oncology guidelines.^20^ These examples illustrate that patient preferences encompass a range of perspectives that include but are not limited to views on treatment options, benefits and risks, and impact not only on health but also on life in general, and may differ across health issues. Importantly, research shows that guidelines that address patient preferences are more likely to be used because the recommendations reflect patient priorities not identified in published evidence upon which guidelines are based, are aligned with patient values that can differ from those of clinicians, and help clinicians engage patients in discussion and shared decision‐making, ultimately leading to higher rates of guideline adherence by patients.^21^, ^22^

However, research shows that many guidelines do not address patient preferences. For example, a 2007 survey of 31 international guideline developers found that 58% included patients on guideline panels and 45% surveyed patients regarding preferences.^23^ An analysis of 137 guidelines published from 2008 to 2013 found that few described patient involvement in guideline development or included preference discussion tools.^24^ Few of 101 American developers evaluated in 2016 required patient involvement on guideline panels (8%), asked patients to review draft guidelines (13%) or offered preference discussion tools in their guidelines (20%).^25^ One reason may be that evidence on how best to identify, incorporate and report patient preferences in guidelines is sparse. A synthesis of research on patient involvement in guideline development or implementation published before 2010 found that few studies offered substantial information about the processes or resources they employed, and largely identified challenges such as tension between patient and clinician priorities.^26^ Similarly, content analysis of methodological handbooks for incorporating patient preferences in guidelines concluded they provided little detail on how to do so.^27^

In the last ten years, there has been increasing emphasis and research on how to achieve patient‐centred care by engaging patients in their own care and in planning and improvement activities that benefit all patients such as developing guidelines; hence, more insight may now be available on how to generate patient preference‐informed guidelines.^28^, ^29^, ^30^ The overall aim of this research was to assemble knowledge that could be widely employed by developers to enhance the implementability of their guidelines as a strategy for supporting guideline use. The specific purpose was to synthesize research published in 2010 or later on how developers can identify, incorporate and report patient preferences in guidelines including processes and determinants (facilitators, barriers) and the potential impact on guideline development processes, guidelines and guideline use.

## 2 METHODS

### 2.1 Approach

We conducted a scoping review using the most recently generated recommended methods comprised of five steps: scoping, searching, screening, data extraction and data analysis,^31^, ^32^ and complied with a reporting checklist specific to scoping reviews.^33^ We chose a scoping review over other types of syntheses because it is characterized by the inclusion of a range of study designs and processes or outcomes, which facilitates exploration of the literature in a given field, reveals the nature of existing knowledge and identifies issues requiring further primary study.^31^, ^32^, ^34^ Similar in rigour to a systematic review, a scoping review does not assess the methodological quality of included studies and does not assume or generate a theoretical stance.^31^, ^32^, ^34^ We did not require research ethics board approval as data were publicly available, and we did not register a protocol.

### 2.2 Scoping

To scope or become familiar with the literature on this topic, we conducted an exploratory search in MEDLINE using Medical Subject Headings: [patient participation/methods and practice guidelines as topic]. The purpose was to peruse examples of potentially relevant studies and, based on that information, inform the development of preliminary eligibility criteria and generate a more elaborate search strategy. CK screened and discussed titles and abstracts with ARG. Together, they drafted eligibility criteria based on the PICO (participants, issue, comparisons, outcomes) framework, which were reviewed and refined by MJA and WBB.

### 2.3 Eligibility

We included studies in which *participants* were adult patients aged 18+, or family members or care partners with or without guideline experience; health‐care professionals of any specialty or setting of care with or without guideline experience; or developers of guidelines including health‐care professionals, managers or staff. The *issue* of interest were studies that described or evaluated the processes and/or impact of identifying, incorporating or reporting patient preferences in clinical guidelines on any procedure (ie preventative, screening and diagnosis) or treatment for any condition. Preferences referred to personal or clinical needs and values for treatment benefits, risks and outcomes.^18^ Studies conducted in all countries and published in English language were eligible. *Comparisons*, referring to data that were collected or units of analysis, included description or evaluation of single or combined processes for identifying, incorporating or reporting patient preferences, or before‐after comparisons, or comparison of two or more processes. Study design included qualitative (ie interviews, focus groups, qualitative case studies, content analysis), quantitative (ie questionnaires, time series, before/after studies, prospective or retrospective cohort studies, trials), multiple or mixed‐methods, or programme evaluation studies. *Outcomes* included but were not limited to exploring participant views, awareness or knowledge about patient preferences or whether/how they should be considered and in what guideline development steps; described processes for identifying, incorporating or reporting preferences; identified determinants (facilitators and barriers) of identifying, incorporating or reporting patient preferences; or assessed the impact of patient preferences on guideline development processes, guidelines and guideline‐related products, or use of guidelines.

We excluded studies if they examined the effectiveness of clinical interventions (tests, procedures, treatment for diseases) rather than approaches for engaging patients or patient preferences in guideline development, did not collect or consider patient preferences or did not pertain to guidelines. We also excluded editorials, letters, commentaries, protocols and meeting abstracts. We excluded paediatric studies where parents function as surrogate decision‐makers, a scenario that differs from direct engagement of patients warranting separate study. Systematic reviews were not eligible, but references were screened to identify eligible primary studies. Guideline development manuals or manuals specific to patient involvement in guideline development were not eligible because we aimed to describe empirical evidence on identifying, incorporating and reporting patient preferences rather than expert opinion or practices and because prior research showed that such manuals offered little guidance.^27^

### 2.4 Searching

We shared eligibility criteria and exemplar studies identified in the scoping step with a medical librarian and jointly developed a comprehensive search strategy (File S1) that complied with the Peer Review of Electronic Search Strategy reporting guidelines.^35^ The search strategy employed Medical Subject Headings and a wide range of keywords in various combinations to identify relevant literature regardless of labels used by authors. Using that strategy, we searched for empirical research studies in MEDLINE, EMBASE, CINAHL and Scopus, and for grey literature in OpenGrey and GreyLit from 2010 to 12 November 2019. We chose 2010 because a prior review that included research published before 2010 yielded little insight^26^ and initiation of efforts by the Guidelines International Network to develop the PUBLIC Toolkit, a compilation of expert opinion and practices for involving patients in guideline development,^36^ following which developers were aware of patient engagement approaches. The references of all eligible studies were scanned to identify additional eligible studies.

### 2.5 Screening

To pilot test screening, CK, YK and ARG independently screened titles and abstracts for the first 25 search results against eligibility criteria and discussed discrepancies and how to interpret and apply the eligibility criteria. Thereafter, CK and YK screened all remaining titles and abstracts. Discrepancies were resolved by ARG. CK retrieved full‐text items, which were screened concurrent with data extraction.

### 2.6 Data extraction

A data extraction form was developed by CK and ARG to collect information on study characteristics including author, publication year, country, study objective, research design, processes used to identify, incorporate or report patient preferences, determinants (facilitators and barriers) and findings. To pilot data extraction, CK and ARG independently extracted data from two articles, and compared and discussed findings to refine the data extraction form. CK and ARG undertook two more iterations of independent extraction and discussion of data from four articles. Then, CK extracted data from all articles, and data tables were independently checked by ARG.

### 2.7 Data analysis

We used summary statistics to report study characteristics (date published, country and research design), guideline topics and the number of studies employing different processes for identifying, incorporating and reporting patient preferences. We described the different approaches for identifying, incorporating and reporting preferences, determinants and impacts as reported in included studies. To further characterize the influence of patient preferences on guidelines, we mapping extracted data on how preferences were incorporated in guidelines to a conceptual framework developed by Armstrong et al that included 10 options: nominate guideline topics, prioritize nominated guideline topics, select guideline development group members, frame guideline questions including select outcomes, create an analytic framework including consideration of benefits and harms, conduct or interpret systematic reviews, generate recommendations, assist with dissemination by endorsing guidelines or by developing patient summaries or preference discussion tools, participate in updating the guideline and take part in evaluating the methods and impact of engagement.^37^

## 3 RESULTS

### 3.1 Search results

**FIGURE 1:**
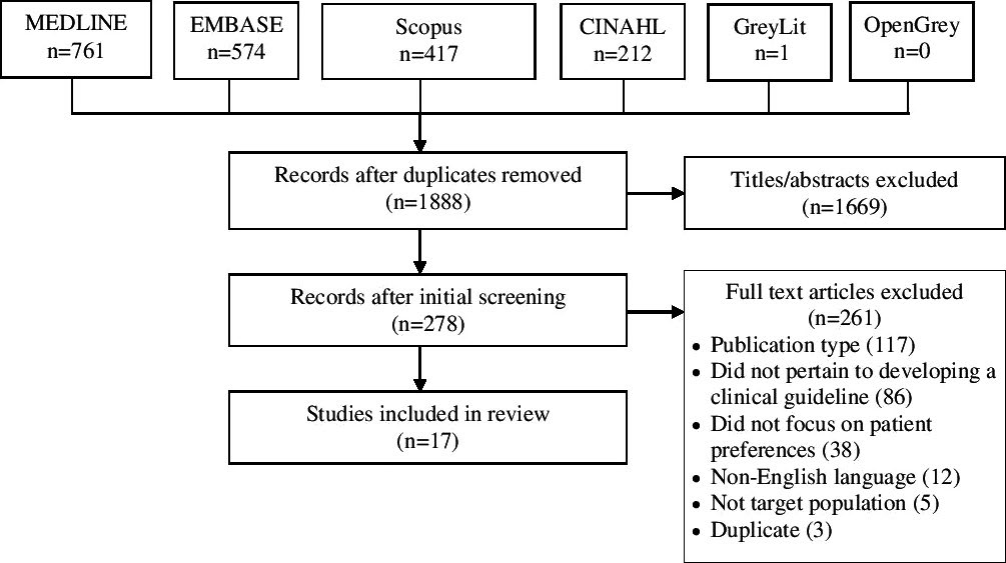
PRISMA diagram

A total of 1,965 studies were identified by searches, of which 1,888 were unique items, and 1,669 were excluded based on screening of titles and abstracts. Among 278 full‐text articles that were screened, 261 were excluded because they were not an eligible publication type (117), did not pertain to developing guidelines (86), did not focus on patient preferences (38), were not English language (12), were not the target population (5) or were a duplicate publication (3). No additional eligible primary studies were identified in systematic review references. A total of 16 studies were eligible for review (Figure 1). Data extracted from included studies are available in File S2.^38^, ^39^, ^40^, ^41^, ^42^, ^43^, ^44^, ^45^, ^46^, ^47^, ^48^, ^49^, ^50^, ^51^, ^52^, ^53^

### 3.2 Study characteristics

Studies were published between 2011 and 2018. Studies were conducted in the United States (5), Netherlands (4), Canada (2), Spain (2) and 1 in each in Australia, England and Finland. The most common research design was qualitative involving document analysis, interviews or focus groups (7), multiple methods studies involving a qualitative component (6), followed by a questionnaire (1), multiple methods involving a systematic review and Delphi consensus process (1) and the RAND consensus technique (1). Clinical topics included multiple health issues (3), arthritis (3), infertility (2), cardiovascular disease (2), cancer, dementia, gynaecologic conditions, kidney disease, palliative care and systematic lupus erythematosus. Two of the 16 studies explored how to best involve patients in guideline development: through focus groups, 20 patients and health professionals said that involvement as a guideline development panel member was the best approach,^46^ and qualitative interviews with 15 researchers, policymakers, guideline developers and patients revealed that the best approach was unknown.^47^ Of the remaining 14 studies, 6 (42.9%) identified patient preferences for the purpose of guideline development,^40^, ^41^, ^42^, ^49^, ^50^, ^53^ and 10 (71.4%) explored various methods for doing so.^38^, ^39^, ^43^, ^44^, ^45^, ^46^, ^47^, ^48^, ^51^, ^52^

### 3.3 Identifying patient preferences

Eight (57.1%) studies employed a single method, and 6 (42.9%) studies employed multiple methods. Two studies included patients as guideline development panellists.^38^, ^39^ Three studies involved patients as panellists and also identified preferences using systematic review,^43^ questionnaire^45^ or systematic review plus Delphi consensus process.^49^ Two studies identified preferences by focus group,^48^, ^52^ and 2 studies employed focus groups plus Delphi consensus process^40^ or questionnaire.^41^ Two studies identified preferences with interviews,^44^, ^50^ and 1 study combined interviews with a questionnaire.^51^ One study employed a questionnaire only,^42^ and 1 study used only a RAND consensus process.^53^

### 3.4 Incorporating patient preferences

Eight (57.1%) studies incorporated preferences in one aspect of guideline development, while 6 (42.9%) studies incorporated preferences in multiple ways. Preferences were used to nominate guideline topics,^48^ prioritize nominated topics,^40^, ^42^, ^44^ inform guideline questions, ^38^, ^49^, ^52^ consider benefits and harms,^38^, ^39^, ^41^, ^43^, ^50^, ^52^ establish the importance of outcomes,^43^, ^50^, ^52^ generate guideline recommendations^45^, ^51^, ^52^, ^53^ and result in the development of a plain language version of the guideline.^52^ Table 1 maps the way that included studies incorporated preferences in guidelines according to the Armstrong et al conceptual framework of engaging patients in guideline development.^37^ No studies incorporated preferences in all or most steps. Included studies incorporated preferences in 6 of 10 possible steps (Table 1). Steps not addressed in included studies were as follows: select guideline development panellists, conduct and/or interpret systematic reviews, update the guideline and take part in evaluating the methods and impact of identifying and incorporating patient preferences. Included studies were too few in number to link any particular single or multiple methods of identifying preferences with incorporating preferences in one or more guideline development steps, or whether particular approaches vary by guideline topic. It did not appear that employing multiple approaches for identifying patient preferences resulted in incorporation of preferences in more steps of the guideline development process. For example, the study that incorporated preferences in the greatest number of guideline development steps (n = 5 steps) employed only a single approach for identifying preferences, a focus group with 15 patients and 8 carers.^52^

**TABLE 1:** Incorporation of preferences in the steps of guideline development

Steps in guideline process^37^	Studies (references)
1. Nominate guideline topics	48
2. Prioritize nominated guideline topics	40, 42, 44
3. Select guideline development group members	—
4. Frame the question(s) (includes considering importance of outcomes)	43, 50, 52
5. Create analytic framework (includes identifying benefits and harms)	38, 39, 41, 43, 50, 52
6. Develop systematic review and form conclusions	—
7. Develop recommendations	45, 51, 52, 53
8. Disseminate and implement recommendations (includes creating alternate versions or accompanying tools)	52
9. Update the guideline	—
10. Evaluate the methods and impact of patient involvement	—

### 3.5 Reporting patient preferences

No studies described if or how the guideline they developed or planned to develop did or would report identified preferences, how preferences influenced the guideline development process or recommendations, or how clinicians can elicit or address preferences in discussions or decision‐making with patients.

### 3.6 Determinants and impact of identifying, incorporating or reporting patient preferences

Determinants are summarized in Table 2. Two studies identified facilitators of involving patients in identifying preferences: both suggested that training in research methods and clinical practice guidelines was needed,^40^, ^46^ and 1 also recommended a combination of in‐person and virtual meetings.^40^ Seven studies identified barriers to identifying patient preferences. Of those, 2 studies noted that it was difficult to find relevant studies that described patient preferences,^43^, ^47^ and 1 reported that patients found it difficult to use an online questionnaire to rank recommendations.^51^ Nearly all other barriers pertained to involving patients as guideline development panellists. Barriers included finding appropriate patients that represented the larger population and not just their own views,^43^, ^46^ institutional review boards that questioned the involvement of patients as panellists,^40^ scheduling meetings at a time convenient for patients,^52^ lack of understanding among patients about what constitutes a preference,^47^ lack of understanding of medical jargon^52^ and patients becoming easily overruled by professional panellists resulting in token involvement.^49^ One study identified a barrier pertaining to incorporating preferences: lack of clarity on how to weight preferences.^47^ No studies explored determinants of reporting patient preferences.

**TABLE 2:** Determinants of identifying, incorporating and reporting patient preferences in guidelines

Study	Barriers	Facilitators
Armstrong^38^	—	—
Li^39^	—	—
Bennett^40^	Adding patient and caregiver stakeholders to the institutional review board protocol Involving them in large conference calls (vs. more personal meetings)	Training in research methods Combination of in‐person and virtual meetings
Goodman^41^	—	—
Pinheiro^42^	—	—
Zhang^43^	Difficult to identify relevant studies that described preferences Information about values and preferences from panel members could be biased and was sometimes difficult to use	—
den Breejen^44^	—	—
Fraenkel^45^	—	—
Hämeen‐Anttila^46^	Difficult to find appropriate persons from the target group who would be capable of representing the larger patient population and not just their own personal experiences and views	Training in clinical practice guidelines
Utens^47^	Understanding of what constitutes a preference Difficult to identify relevant studies that described preferences The weight to give patient preferences	—
Pittens^48^	—	—
Serrano‐Agu^49^	Patients holding their own when facing a team of professionals Becoming easily overruled by professionals resulting in tokenism	—
Garcia‐Toyos^50^	—	—
Den Breejen^51^	Users found it difficult to find and use the website (questionnaire to rank guideline questions) They did not fully understand the purpose of the website (to rank recommendations based on preferences)	—
Tong^52^	Difficult to achieve an adequate attendance rate as some participants were unable to attend at the last minute Medical jargon	—
Musila^53^	—	—

### 3.7 Impact of identifying, incorporating or reporting patient preferences

Although 10 studies aimed to explore methods for identifying preferences, beyond anecdotally stating how preferences did or would influence guideline development steps, few studies explicitly evaluated the processes or impact of identifying or incorporating patient preferences. Three studies found that patients and clinicians nominated^48^ or prioritized different topics.^42^, ^44^ One study by Armstrong et al thoroughly described the impact of having included patients as guideline development panellists.^38^ That study compared guideline questions and key benefits and harms identified by two panels, one with and one without patient representatives. Patient representatives shaped how discussions were conducted, broadened the scope of discussions, described the personal impact of disease and impacted how physicians viewed the topic and patient involvement. Patient representatives described issues not raised by participating physicians, identified patient‐relevant outcomes and contributed to discussions of how future recommendations should be framed. Patient representatives also participated in crafting of plain language guideline questions, suggested a broad target audience for the guideline and identified that patient preferences regarding this topic will vary, all issues with dissemination and implementation implications.

## 4 DISCUSSION

This study identified few studies published since 2010 on approaches for generating guidelines that reflect patient preferences. Of the 16 included studies, 10 aimed to evaluate methods but were largely anecdotal; only one study empirically assessed the impact of involving patients as members of guideline development panels on establishing guideline questions. Studies employed a variety of single and multiple approaches to identify preferences and most often incorporated preferences in identifying, prioritizing or formulating guideline questions; identifying treatment benefits and harms; and prioritizing or informing guideline recommendations.

This study is unique from other research on patients' preferences and guidelines. Prior research explored what patients or the public know about and expect from clinical practice guidelines.^54^, ^55^ A systematic review that included 20 studies published from 1999 to 2017 found that few developed or assessed the properties of questionnaires designed to measure patient preferences.^56^ A conceptual review of select literature identified broad steps of guideline development in which to engage patients,^57^ not unlike work by Armstrong et al^37^ An international expert working group representing a wide range of stakeholders and disciplines generated consensus on nine broad approaches for engaging patients in health research, and policy or regulator decision‐making that included patient perspective, engagement, transparency, representation, multiple inputs, support, expertise, resources and monitor.^58^ Content analysis identified little inclusion of patient preferences in guidelines on implantable cardioverter defibrillator therapy^59^ or guidelines on cardiac rehabilitation or depression.^60^ Thus, subsequent to 2010, this is the only systematic synthesis of empirical research on how to identify, incorporate and report patient preferences in guidelines.

Given that guidelines are more likely to be implementable and used when informed by patient preferences,^21^, ^22^ lack of inclusion of preferences in guidelines^23^, ^24^, ^25^, ^59^, ^60^ and recognized lack of guidance on how to do so,^26^, ^27^ this study identified a persistent paucity of knowledge in this area and revealed multiple ideas for on‐going research to address this knowledge gap. By comparing approaches for incorporating preferences in guidelines with the Armstrong et al conceptual framework,^37^ we identified numerous ways that preferences to date have not, but could in future influence guidelines. However, this study revealed lack of evidence on how to best do so^26^, ^27^, ^46^, ^47^ and, as the most current and comprehensive synthesis of determinants, revealed numerous barriers that may be challenging developer efforts. Prior research involving interviews with 30 developers from 7 countries found that developers perceived that it was important to develop implementable guidelines but lacked necessary resources including funding and staffing.^61^ This underscores the need for developers to direct limited resources to the most useful approaches, particularly given that a comparison of methods for identifying preferences found that patient consultation using a three‐round web‐based Delphi survey identified the same lupus erythematosus guideline questions as either synthesis of published patient preferences research or including patients on a development panel, potentially obviating the need for multiple approaches.^49^ This study revealed three knowledge gaps that should form part of an ongoing research agenda: one, an insufficient volume of studies identified which single or multiple approaches for identifying preferences result in better incorporation of those preferences in guidelines, or whether approaches must vary by guideline topic; two, no studies offered insight on how to report patient preferences in guidelines; and three, and few studies identified barriers associated with approaches to identify preferences other than involving patients as panelists. Until that research becomes available, a review of patient engagement in other forms of health service planning or improvement could provide insight on approaches that could be applied to the development of guidelines.

The strengths of this study include use of rigorous scoping review methods^31^, ^32^ and compliance with standards for the conduct and reporting of scoping reviews and search strategies.^33^, ^35^ We searched the most relevant databases of medical literature and employed the same rigorous methods to search for and screen grey literature. We mapped findings to an established conceptual framework of patient engagement in the steps of guideline development as a means of further interpreting the results.^37^ Several limitations must also be noted. Our search was limited to English language studies, so we may not have included relevant studies published in other languages. The search strategy may not have identified all relevant studies, or our screening criteria may have been too stringent. The included studies provided limited and anecdotal details on approaches, barriers and impacts; thus, on‐going research is warranted.

## 5 CONCLUSIONS

Despite the recognized importance of generating guidelines informed by patient preferences, little research over the last decade has generated guidance on how best to identify, incorporate or report patient preferences. This review identified numerous ways that preferences have not, but could have influenced guidelines, and thoroughly summarized facilitators and barriers, knowledge needed by developers to expand and improve their processes. Further research is needed to establish the single or multiple approaches that lead to the incorporation of preferences in the full range of guideline development steps and explicit reporting of those preferences in guidelines.

## CONFLICT OF INTEREST

The authors have no conflicts of interest to declare.

## Supporting information

Supplementary Material

Supplementary Material

Supplementary Material

## Data Availability

Data are available in article supplementary material.
