# Supplementary material for: How to identify, incorporate and report patient preferences in clinical guidelines: A scoping review
Source: Health Expect. 2020 Jul 12;23(5):1028–36. doi: 10.1111/hex.13099 (PMC7696279; doi:10.1111/hex.13099)
Supplement: Supplementary file 1 — Supplementary Material [file HEX-23-1028-s001.docx]

Additional File 1. Search Strategy

Database(s): **Ovid MEDLINE: Epub Ahead of Print, In-Process & Other Non-Indexed Citations, Ovid MEDLINE® Daily and Ovid MEDLINE®** 1946-Present

| **#** | **Searches** | **Results** |
| --- | --- | --- |
| 1 | exp GUIDELINE/ | 30259 |
| 2 | guidelines.mp. | 339871 |
| 3 | guideline.mp. | 92243 |
| 4 | 1 or 2 or 3 | 384315 |
| 5 | exp Patient Participation/ | 22000 |
| 6 | patient engagement.mp. | 1619 |
| 7 | exp Patient-Centered Care/ | 16013 |
| 8 | exp Patient Preference/ | 5945 |
| 9 | 5 or 6 or 7 or 8 | 43242 |
| 10 | 4 and 9 | 2840 |
| 11 | exp Patients/ | 55870 |
| 12 | patient.mp. | 2365509 |
| 13 | patients.mp. | 5072309 |
| 14 | 11 or 12 or 13 | 6131113 |
| 15 | engage.mp. | 37449 |
| 16 | engagement.mp. | 45578 |
| 17 | 15 or 16 | 79328 |
| 18 | 14 and 17 | 20900 |
| 19 | involve.mp. | 136351 |
| 20 | involvement.mp. | 399882 |
| 21 | 19 or 20 | 528508 |
| 22 | 14 and 21 | 196064 |
| 23 | 10 and 18 | 198 |
| 24 | 10 and 22 | 226 |
| 25 | limit 24 to english language [****CM search strategy****] | 214 |
| 26 | guideline/ or practice guideline/ | 30259 |
| 27 | guidelines as topic/ or practice guidelines as topic/ | 138213 |
| 28 | (guideline or practice guideline).pt. | 30259 |
| 29 | guideline*.au,ti,ab,kf. | 280000 |
| 30 | or/26-29 [***Guideline terms***] | 371843 |
| 31 | Program Development/ | 26326 |
| 32 | 30 and 31 [***Guideline development results***] | 2220 |
| 33 | (guideline* adj3 develop*).ti,ab,kf. | 15560 |
| 34 | 30 or 33 [****Guideline development terms****] | 371843 |
| 35 | exp Patient Participation/ or exp Patient-Centered Care/ or exp Patient Preference/ | 42091 |
| 36 | ("patient* involve*" or "patient* engage*" or "patient* participat*").ti,ab,kf. | 17305 |
| 37 | or/35-36 [****Patienet participation terms****] | 56907 |
| 38 | "attitude of health personnel"/ | 108875 |
| 39 | practice patterns, nurses'/ or practice patterns, physicians'/ | 53154 |
| 40 | professional-patient relations/ or nurse-patient relations/ or physician-patient relations/ | 124077 |
| 41 | or/38-40 [***health professional terms****] | 263640 |
| 42 | 34 and 37 and 41 [***Base clinical set****] | 777 |
| 43 | 42 not 24 [***EU Unique results from revisions****] | 718 |
