# Supplementary material for: How to identify, incorporate and report patient preferences in clinical guidelines: A scoping review
Source: Health Expect. 2020 Jul 12;23(5):1028–36. doi: 10.1111/hex.13099 (PMC7696279; doi:10.1111/hex.13099)
Supplement: Supplementary file 2 — Supplementary Material [file HEX-23-1028-s002.docx]

Additional File 2. Data extracted from included studies

| Study | Guideline topic | Research Design  Participants | Objective | Patient Preferences | | | Findings |
| --- | --- | --- | --- | --- | --- | --- | --- |
|  |  |  |  | Identify | Incorporate | Report |  |
| Armstrong 2018 United States [38] | Amyloid positron emission tomography (PET) imaging in patients with or at risk for dementia | Qualitative – observation of two simultaneous guideline development panels at a one-day meeting, one panel including patients (experimental group) and one physicians alone (control). Content analysis was used to examine meeting output transcripts  18 participants two methodologists, two facilitators, one dementia content experts, two dementia imaging content experts, seven members of the guideline subcommittee with and without dementia expertise and four lay participants | To investigate the effect of patient involvement on guideline question formation | Participants received training regarding the topic and guideline development at the meeting and split off into experimental and control groups for discussion. The two groups were asked to develop questions for the amyloid PET guideline, identify relevant benefits and harms, and craft patient-language versions of proposed questions | Guideline questions  Benefits/harms | --- | Proposed guideline questions, benefits, and harms were largely similar between groups, but only the experimental group proposed outcomes relating to development of cognitive impairment at specific time points, rate of progression and developed patient versions of the questions, demonstrating the impact of having included patients in this process |
| Li  2018  Canada  [39] | Management of venous thromboembolism | Qualitative – content analysis of meeting transcripts  48 members from 12 countries, 8 of whom were patients, met to develop five guidelines | To explore how panelists used the Grading of Recommendations Assessment, Development, and Evaluation (GRADE) criteria when formulating guideline recommendations | Panel chairs typically consulted patient representatives as a source for assessment of patient vales and preferences when patient values were the topics of interest. A patient representative from one of the panels was asked for their perspective on doing an ultrasound to assess for silent deep vein thrombosis and the patient vocalized the benefits and harms for the procedures. | Benefits/harms | --- | Differences in GRADE criteria preferences between patients and clinicians were not reported, though it appears that patients and clinicians similarly formulated recommendations |
| Bennett 2017 United States [40] | How to balance benefits and harms of treatment for people with multiple chronic conditions including cardiovascular disease | Multiple methods – (1) Two-round Delphi process to prioritize potential topics involving clinicians, patients and carers (number not specified)  (2) Four 90-minute qualitative focus groups with 27 patients and carers to elicit views on two important research questions and relevant patient-important outcomes | Identify two high-priority clinical questions related to multiple chronic conditions and identify patient-important outcomes for those questions | From 130 potential questions, the Delphi process prioritized 12 questions, then in focus groups, patients and carers further prioritized 2 questions and identified benefits and harms related to the questions | Prioritize guideline topics | --- | Two questions pertained to optimal blood pressure goals and diabetes medication management. Patient-important outcomes were:   - Physical function and energy - Emotional health and well-being - Avoidance of treatment burden, side effects and risks - Interaction with providers and health care system - Prevention of adverse long-term health outcomes |
| Goodman  2017  United States  [41] | Perioperative management of antirheumatic medication in patients with rheumatic diseases undergoing elective total hip arthroplasty or total knee arthroplasty | Multiple methods – qualitative focus group among 11 panelists who were patients with rheumatoid arthritis who had undergone hip or knee replacement (10 female, mean age 47); panelists then anonymously voted on the importance of 7 potential guideline questions | To describe patient contributions to the development of the antirheumatic medication guidelines | The patient panel completed 8 hours of training on guideline development by webinar, then took part in a one-day meeting where they reviewed the evidence and were asked to discuss the relative importance of infections, infrequent events possibly linked to continued immunosuppressant disease‐modifying antirheumatic drug and biologic agent therapy, compared to the importance of flares of disease that occur frequently after joint replacement and may be linked to withholding the medications | Benefits/harms | --- | Panelists uniformly attached far greater weight to the possibility of infections if medications were continued despite the greater likelihood of a flare if the medications were stopped. Flares represented a “known risk” that they could control and that in particular can usually be treated.  Panelists decided to continue or withhold medication for each of the 7 questions; decisions matched those of the clinician panel; however, panelists stressed that the recommendations should be individualized to each patient |
| Pinheiro  2017  United States  [42] | Guidance for patient-clinician discussions about molecular testing to inform treatment decisions for cancer patients | Multiple methods – qualitative observation of patient-clinician consultation, and questionnaire to prompt choice of discussion topics and preferred format for information about molecular testing  66 patients (46 women, median age 62) with various types of cancer and 27 physicians based at one hospital | To identify physician and patient preferences for discussion topics related to molecular testing | Patients were asked to choose 8 topics they most wanted to discuss from a list of 18 potential topics, and preferred source of information: written information, short video, website, from a physician or nurse, or from another patient. Questions pertained to the domains of consent, treatment and results, additional information about the tests, and follow-up support | Prioritize guideline topics | --- | Patients and clinicians differed in question priority and preferred information format  Patients' top 2 preferred topics were the benefits of testing (88%) and how testing determines treatment (88%). They also chose 10 additional topics: implications for family (71%), whether the test indicates seriousness of disease (68%), test purpose (64%), incidental findings (56%), explanation of cancer genetics (53%), how the test is done (46%), limitations (44%), explanation of biomarker (42%), risks (42%), and an uninformative result (38%). Patients most frequently selected a discussion with their nurse or physician (85%) and written information (67%) as their preferred methods for receiving information.  Physicians' top choices were: how the test determines treatment (100%), test purpose (93%), and benefits (89%). Physicians also chose limitations (70%), explanation of biomarker (63%), cost (59%), how the test is done (56%), risks (56%), and prognostic information (52%). The physicians' preferred aids to communication were pamphlets (67%), followed by a website explaining key facts (44%), patient video (41%), and scripts for them to use (26%). |
| Zhang  2017  Canada  [43] | Patient preferences for outcomes relevant to 22 guidelines on a variety of topics | Multiple methods – systematic review, discussion with clinician and patient guideline development panel members (number of panelists and characteristics not reported) | To provide an overview of a process for systematically incorporating values and preferences in guideline development | Primary studies included in systematic reviews identified patient preferences with utility-based estimates, surveys, or qualitative research. The number, type and findings of those studies were not reported. | Importance of outcomes  Benefits/harms | --- | Outcome preferences were identified in published research, and from patient and clinician guideline development panelists and were said to influence the rating of recommendations as weak or strong; however, findings were anecdotal so not clear if patient and clinician preferences differed or how they influenced specific guideline recommendations |
| den Breejen  2016 Netherlands [44] | Multidisciplinary guideline on management of infertility | Qualitative – interviews with 12 infertile couples and focus groups with 17 clinicians (7 female): general practitioners, urologists, gynaecologists, psychologists) | To assess how patient preferences contributed to the scoping phase of guideline development | Infertile couples identified 32 issues and clinicians identified 23; infertile couples identified 8 issues not mentioned at all by clinicians. | Prioritize guideline topics | --- | Patients and clinicians prioritized different key elements of care; which influenced decisions about guideline topics |
| Fraenkel  2016  United States  [45] | Treatment of rheumatoid arthritis | Questionnaire – voting for or against recommendations and rating of recommendations as strong or conditional was compared between a 10-member patient panel (7 women) and a panel comprised of 9 physicians and 2 patients | To comparing the selection and grading of recommendations between a patient-only panel with a panel largely comprised of physicians | Patients completed 8 hours of webinar training on guideline development, then took part in a three-day meeting that included voting on recommendations. | Guideline recommendations | --- | The patient panel developed 16 of the 18 recommendations presented to them; for 13 of the 16 recommendations, patients recommended the same course of action as did the largely physician panel. Decisions differed based on how the two panels valued the balance between benefits and harms. It appears that patients influence the formulation of recommendations because their values for outcomes differ from clinician values |
| Hämeen-Anttila  2016  Finland  [46] | Explore patient involvement in development of guidelines on many topics | Qualitative – focus groups with 20 patients and health professionals from 14 patient organizations (number of patients not reported) | To identify ways to involve patients in guideline development and related challenges | While surveys were said to be the most common way to gather patient preferences, participants said it was important to gather input from groups, rather than individual patients, and suggested they be involved as panel members | --- | --- | Challenges of involving patients on guideline development panels were identifying patients, offering convenient times for them to participate, and sharing information with them in a clear manner |
| Utens  2016  Netherlands  [47] | Explore patient involvement in development of guidelines in general | Qualitative – 15 interviews with researchers (7), policy makers and guideline developers (4) and patients (4) | To explore how to integrate patient preferences in pharmaceutical coverage decisions and clinical practice guidelines, and associated facilitators and barriers | Participants agreed that patient preferences should be incorporated in guidelines but cited lack of guidance on how, and noted numerous challenges including time, funding and staff to do so | --- | --- | It is unclear how best to integrate patient preferences in guideline or balance patient preferences with other decision criteria or priorities |
| Pittens  2015  Netherlands  [48] | Guideline on return to work following gynaecological surgery | Qualitative – focus groups with 21 women who had gynaecological surgery | Explore patient involvement in development of guidelines on incidental or non-threatening diseases | Patients were asked about perceived problems, needs and preferences both before and after surgery, and other concerns regarding after-care and counselling in resumption of work or related activities. Issues identified by patients formed the basis of a Delphi study among health care professionals to choose topics upon which the guideline recommendations would be based | Nominate guideline topics | --- | Topics selected by professionals included several issues brought up by patients.” Those topics pertained to activities of daily living (i.e. taking a bath, jumping) whereas professionals focused on movements like walking, lifting, bending, etc.  Specific details about how patient preferences influenced recommendations were not reported. There appear to be differences in the topics prioritized by patients and clinicians |
| Serrano-Aguilar  2015  Spain  [49] | Management of Systemic Lupus Erythematosus | Multiple methods –systematic review (22 studies in 24 articles 1988-2011) of patient perceived health care needs; three-round Delphi process involving 102 patients (93 women) to establish needs; and a patient was included on the 16-member guideline panel | To incorporate patient perspectives in the design of a clinical practice guideline | Health care needs and concerns about health care quality were blended and shared with the guideline development panel who used that information to generate key questions (process/methods not reported, nor how patient panelist was involved) | Guideline questions | -- | Most relevant needs across the systematic review and Delphi were physical, psychological, familial, and socio-economic. Dissatisfaction with health care services was mainly due to unmet information needs and limited access to care. These concerns appear to have informed questions upon which the guideline was based. |
| Garcia-Toyos  2014  Spain  [50] | Opioid analgesic use in terminal care | Qualitative – interviews with 22 terminal patients and 20 caregivers | To identify the values and preferences of terminal patients and their caregivers regarding treatment with opioids and on the desirable outcomes | Interviews generated information that was to be considered when developing the guideline (how was not reported) | Importance of outcomes  Benefits/harms | --- | Participants expressed interest in the use of opioids for pain alleviation and noted adverse digestive effects that can result in abandoning its use. They were unaware of potential side effects, but desired more information and more involvement in treatment decision-making |
| Den Breejen  2012  Netherlands  [51] | Multidisciplinary guideline on management of infertility | Multiple methods – qualitative interviews with 12 infertile patients to generate recommendations; and online questionnaire of 298 infertile patients to rank top 5 recommendations in each of 5 sections on fertility care | To identify potential guideline questions informed by patient needs and preferences, and evaluate an online tool (wiki) as a means of collecting preferences | Interviews generated 289 recommendations; from these, patient ranking prioritized 21 recommendations. All 21 were incorporated into the guideline | Guideline recommendations | --- | Top-ranked recommendations were organized as general care, general practice care, gynecologic care, urologic care and laboratory issues. Barriers to use of the online tool were identified by survey of 45 patients and interviews with 3 of those; barriers pertained to format and content of the web site, and ability to find it. |
| Tong  2012  Australia  [52] | Management of early stage chronic kidney disease | Qualitative – workshop discussion involving small group brain-storming exercises among 15 patients and 8 carers | To explore how to involve consumers in guideline topic and outcome selection, and the impact of that involvement on the guideline | Participants generated views on patient and carer perspectives on living with early stage chronic kidney disease; the benefits and harms of tests, treatments and interventions; topics or questions they thought should be included in the guideline; outcomes they perceived as important; and what they thought clinicians need to understand about patient and carer perspectives | Guideline questions  Outcomes of importance  Benefits/harms  Guideline recommendations  Development of a plain English version of the guideline | --- | Recommended topics were: patient education, monitoring, nutrition and exercise, managing fatigue, medication side-effects and interaction, emotional and financial support for patients and carers, and health care services (communication, continuity of care). Prioritized outcomes treatment effectiveness, survival, physical symptoms and side-effects, costs, emotional status, and the social impact, long-term effects, accuracy, and discomfort and pain of tests. |
| Musila  2011  England  [53] | Referral to specialists by general practitioners for patients with osteoarthritis of the knee | Consensus process using RAND appropriateness criteria approach – guideline panels comprised of 3 patients, 3 general practitioners, 3 orthopedic surgeons and 3 other health care professionals | To develop a referral guideline for patients with chronic knee pain that explicitly incorporates patients’ preferences. | Panelists completed a questionnaire to rate agreement with 12 recommendations based on case scenarios that varied by patient age, symptom severity, body mass, co-morbidity and patients referral preference. Results were shared at an in-person meeting where panelists could re-rate the scenarios (patient panelist ratings not reported) | Guideline recommendations | --- | Ratings of referral appropriateness for the 108 scenarios were strongly influenced by symptom severity and patient preferences for referral, and patient preference depended on severity, or referral was desired when symptoms were moderate or severe rather than mild. |
